# Integrated omics approaches with non-thermal food fortification: pathways to personalized nutrition solutions

**DOI:** 10.3389/fnut.2026.1778056

**Published:** 2026-02-27

**Authors:** Saleh A. Alsanie

**Affiliations:** 1 Department of Basic Health Sciences, College of Applied Medical Sciences, Qassim University, Al-Melida, Saudi Arabia; 2 Department of Clinical Nutrition, Medical City, Qassim University, Al-Melida, Saudi Arabia

**Keywords:** bioavailability, food fortification, metabolomics, microbiome, multi-omics, non-thermal processing, nutrigenomics, personalized nutrition

## Abstract

The integration of multi-omics technologies with non-thermal food processing represents a paradigm shift from population-based to precision nutrition interventions. This review synthesizes current advances in genomics, transcriptomics, proteomics, metabolomics, and microbiomics alongside emerging non-thermal technologies, including high-pressure processing, pulsed electric fields, cold plasma, ultrasound, and supercritical fluid extraction, to enable the development of personalized fortified foods. Non-thermal processing offers distinct advantages by preserving heat-sensitive nutrients, enhancing bioavailability through matrix modification, and supporting innovative encapsulation systems that overcome limitations of conventional thermal methods. Multi-omics approaches provide insights into genetic polymorphisms, metabolic phenotypes, and microbiome profiles that influence nutrient metabolism, thereby informing targeted fortification strategies for individuals and subpopulations. We examine nutrient-gene interactions, the impact of non-thermal processing on food matrices and fortificant stability, and the integration of complex omics datasets through systems biology. Key challenges include industrial scalability, harmonization of omics data interpretation, regulatory frameworks for personalized products, equitable access, and genetic data privacy. Emerging opportunities involve artificial intelligence for predictive modeling, biosensor-based monitoring, blockchain-enabled traceability, and convergence with precision medicine. This review provides a comprehensive framework to guide researchers, food technologists, healthcare professionals, and policymakers in advancing omics-guided, non-thermally processed fortified foods as innovative strategies for addressing malnutrition, preventing chronic disease, and promoting optimized health across diverse populations.

## Graphical abstract

**Figure:**
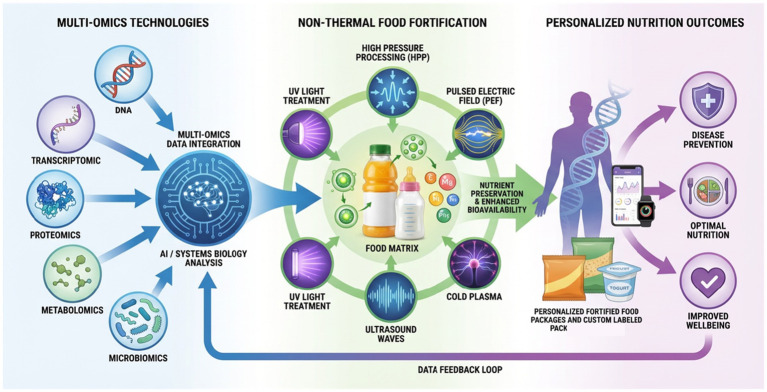

## 1 Introduction

Food fortification has been identified as one of the most cost-effective public health intervention aimed at reducing micronutrient deficit in different populations around the world (1). Fortification of staple foods with essential vitamins and minerals or bioactive compounds is a key approach to addressing important nutritional gaps, especially in vulnerable groups such as infants, pregnant women and those living in resource-poor settings (2). The world market for functional and fortified foods has grown rapidly from USD 280.7 billion in 2021 due to rising consumer awareness on the role of preventive nutrition coupled with an escalating burden of diet-related chronic diseases (3). Programmatic fortification interventions have shown an impact on population-level micronutrient status (4). However, conventional fortification strategies have ongoing problems related to poor nutrient stability following processing, low bioavailability, and an inability to accommodate large interindividual variability in response to dietary nutrients requirements and metabolic responses (5).

Although conventional thermal processing is necessary for safety and shelf life, it represents a major challenge to maintain the stability of heat sensitive micronutrients and phytochemicals incorporated during fortification (6). High temperature applications such as pasteurization, sterilization and processes of spray drying or extrusion, can result in a number of degradation reactions which affects the expected nutritional value in fortified products (7). Vitamin C, folate, thiamin, and B-group vitamins are particularly sensitive to heat degradation due to oxidation and Maillard reactions whereas thermal treatment cause structural destruction of the polyphenolic compounds including anthocyanins (8). The amount of nutrient loss is matrix specific and it depends on the processing conditions such as temperature, time, availability of oxygen and humidity. In addition to direct chemical degradation, thermal processing can adversely affect the food matrix by diminishing both bioaccessibility and subsequent bioavailability (9). The denaturation of proteins and the oxidative degradation of lipids due to high thermal treatment may trap or conjugate supplemented micronutrients, thus inhibiting their release and absorption in the gastrointestinal tract. Such thermally induced losses have broadened the gap between theoretical fortification values and actual nutrient available at the consumer level, resulting in loss of program impact efficacy or additional supplementation that will increase costs and regulatory burden (10).

In addressing these issues, non-thermal food processing technologies have been developed as alternative methods that not only kill microorganisms and induce desired changes, but also help in maintaining heat-labile nutrients to a larger extent (11). High Pressure Processing (HPP) inactivates microorganisms using high pressure up to 400–600 MPa at room temperature, and has shown better retention of vitamins, polyphenols and sensory factors in fortified beverages than thermal pasteurization treatment (12). The technology works through pressure mediated killing of microbial membranes without creating heat, and hence the added fortificants are retained structurally and functionally during processing, minimizing thermal degradation and chemical transformation, while remaining fully subject to mandatory ingredient labeling and regulatory disclosure requirements (13). Pulsed Electric Field (PEF) involves the application of short pulses of high-voltage electricity that leads to the reversible permeabilization of the cell membrane, markedly increasing extraction of phytochemicals and inactivating microorganisms with insignificant thermal effect (14). Cold plasma treatment, which is produced by the ionization of gasses at nearly room temperature, has been reported for potential modification of food surfaces and improving mineral bioavailability using surface oxidation without any degradation due to heat (15). Ultrasound processing utilizes high-frequency sound waves to produce cavitation and mechanical abrasion that enhances extraction at elevated levels of added micronutrient bioaccessibility, allowing for controlled matrix modification (16). Together, these non-thermal technologies provide a variety of options for the production of fortified foods with better nutrient retention and bioavailability as well as sensorial properties (16).

Advancement of omics technologies, including genomics, transcriptomics, proteomics, metabolomics and microbiomics has transformed understanding of complex interactions among diet, individual biology and health outcomes (17). Meanwhile, nutrigenomics has uncovered the genetic polymorphisms dramatically modulate the individual need for certain micronutrients and the metabolism to nutritional ingredients and diet-related diseases (18). Transcriptomic and proteomic technologies permit the establishment of patterns of gene expression in response to nutrients or changes in protein levels following consumption of bioactives, providing information on how added compounds function on a physiological level. Metabolomics records dynamic biochemical fingerprints from dietary exposures, providing sensitive biomarkers for evaluating intake of fortified foods and monitoring metabolic responses (19, 20). Microbiomics has become an important dimension in personalized nutrition, since the microbiome plays a central role in nutrient biotransformation, production of bioactive metabolites and modulation of host metabolism and immunity (21). Moreover, these multi-omics platforms, which are being increasingly driven by machine-learning algorithms can also be integrated to even predict personal response to dietary-interventions and population stratification according to individual’s unique nutritional requirements and metabolic phenotypes (22). This omics-based transition from generalized population-wide advice toward precision nutrition approaches to fortification has the potential to ensure that the choice, level, and delivery format of the fortificant is matched more closely to individual biological need (23).

The integration of non-thermal fortification technologies and omics-based personalized nutrition provides a transformative tool to tackle the perennial problems in nutritional public health (24). By maintaining the structural architecture and bioactivity of supplemented micronutrients during processing, non-thermal technologies can deliver the required substrate bioavailable, stable fortificants to enable precision nutrition strategies to deliver the health impact (25). Conversely, omics-based knowledge of each individual’s genetic background, metabolic potential, and microbiome community can guide the development of customized fortified foods for subpopulations or even individuals beyond one-size-fits-all as in typical fortification programs (26). The integration depends on the coordinated progress of multiple fields including food engineering to optimize the non-thermal process parameters, analytical chemistry for the validation of nutrient retention and bioaccessibility, bioinformatics that will enable multi-omics datasets integration, and regulatory science with regard to personalized fortified products frameworks (27). Despite substantial advances in non-thermal food processing and omics sciences, important controversies and research gaps remain. The suitability of specific non-thermal technologies for different fortificants is fragmented, particularly regarding long-term stability, matrix-dependent bioavailability, and inter-individual metabolic responses. In parallel, although multi-omics approaches have improved understanding of nutrient-gene-microbiome interactions, their translation into industrial food fortification strategies remains largely conceptual, with unresolved challenges related to scalability, data integration, regulatory approval, and ethical governance.

Accordingly, a critical gap exists at the intersection of non-thermal fortification and omics-guided personalized nutrition, as most existing reviews address these domains in isolation rather than as an integrated system. This review addresses this gap by critically evaluating their convergence, guided by the following review questions: (i) Which fortificants are most compatible with specific non-thermal technologies in terms of stability and bioavailability? (ii) How can multi-omics approaches inform fortificant selection, dosage, and food matrix design at an industrial scale? (iii) To what extent is the implementation of precision fortification feasible within current regulatory frameworks? By addressing these questions, this review provides a focused framework for advancing omics-informed, non-thermally fortified foods toward practical precision nutrition applications.

## 2 Non-thermal technologies in food fortification

### 2.1 High-pressure processing

**Figure 1:**
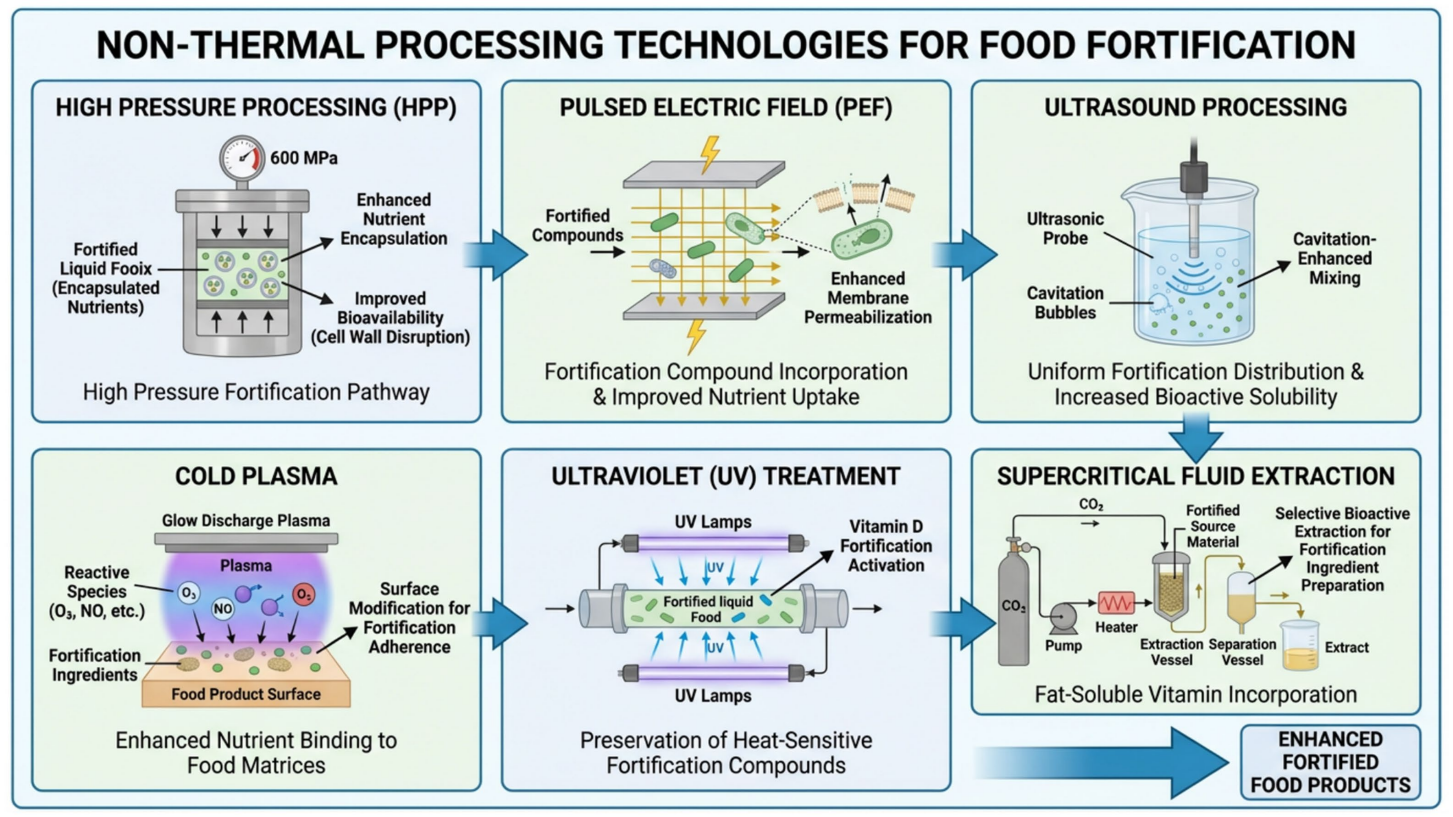
Non-thermal processing technologies for food fortification.

High pressure processing (HPP) is a non-thermal technology with the potential of incorporating nutrients and can preserve heat-sensitive nutrients to a greater extent than traditional thermal processes shown in Figure 1. HPP can be used for non-thermal inactivation of microorganisms and modification of enzymes, having less effect on the degradation of bioactive compounds by applying a uniform hydrostatic pressure in the range 100–600 MPa over short periods (28). The process is performed through volume compaction and macromolecule structuring, which contributes to the killing of the microbe but does not destroy low-molecular-weight food components unless such are evolved (28). Advantages of vitamins retention under HPP conditions are well defined. For water-soluble vitamins, stability is wide-ranging: riboflavin was better stabilized by ultra-high-pressure homogenization (UHPH) with a reduction of degradation levels of 50% and improved protection to photolytic and oxidative stress. Vitamin C typically exhibits little immediate loss after HPP, but storage stability is matrix dependent (29). The results for fat-soluble vitamins and provitamins are even more positive. For example, *β*-carotene in barley in non-dairy milk subjected to pulsed HPP treatment at 100 MPa was retained but thermal treatment at 80 °C caused 20% loss (30). Similarly, smoothies processed at 450–600 MPa for 3 min reported enhanced *β*-cryptoxanthin, *α*-carotene and β-carotene, and also lutein, zeaxanthin as well as vitamin E compared to non-pressed formulations (31).

Additionally, HPP-induced structural changes improve mineral bioavailability. The High-pressure impregnation (HPI) of staple grains such as rice allows for fortificant transfer through pressure-driven mass transport and the cold-condition starch gelatinization (32). These alterations would allow for greater permeability into the cell wall and dissolution of minerals, however potential interactions with endogenous enzymes, as well as matrix binding should be accounted for bioavailability predictions (33). Another benefit is the retention of the bioactive compounds. In the moderate range (HPP) from 100 to 300 MPa and under pulsed condition, HPP disrupts cell structure making phenolics and flavonoids more available for extraction without causing cellular morphology damages. HPP-treated barley particles with optimal treatment at 300 MPa and 40 °C have higher phenolic, flavonoid contents, and antioxidant activities than the thermally treated ones (30). Preservation of carotenoids in fruit beverages and enhanced antioxidant capacity in UHPH-treated milk are additional indications of the HPP technology’s effectiveness (29).

Recent applications comprise of plant-based beverages fortified with *β*-carotene, carotenoid-rich smoothies and grains loaded with micronutrients. The combination of pressure intensity, number of pulses and temperature allows the simultaneous optimization for microbial safety and (sensory) quality (34). Moderate pressure pulsing along with low thermal loading lead to preservation of labile bioactives and improved extractability, establishing HPP as a pivotal technology in personalized nutrition via integrated omics approaches (35).

### 2.2 Cold plasma technology

Cold plasma technology has emerged as a potential non-thermal technology for food fortification, with a potential for enrichment of nutrients and surface functionalization without thermal degradation (36). As a cold plasma that is partially ionized with reactive species, electrons, ions and UV photons, operates at near room temperature and is therefore ideal for incorporation of nutrients which may be heat sensitive (37). Its systems can be largely divided into atmospheric-pressure and low-pressure conditions, each of which has its own advantages (38). Atmospheric-pressure sources, including dielectric barrier discharge (DBD), atmospheric plasma jet (APPJ) and corona discharge operate at room pressure at frequencies 0.05–500 kHz. DBD systems create high-energy species with low energy input and modifiable design, but the flexibility could be limited by electrode confinement (39). APPJ systems emit reactive species into ambient air, which allows direct treatment of different substrates, and low-pressure plasma gives better control over the generation of reactive species as well as depth of penetration, especially in grain and cereal fortification (40).

The antimicrobial effect of cold plasma is a result of synergy actions involving reactive oxygen (ROS) and nitrogen species (RONS), such as OH radicals, hydrogen peroxide (H₂O₂), ozone (O₃), nitric oxide (NO), and nitrogen dioxide (NO₂) that damage membrane and deoxyribonucleic acid (DNA). Ultraviolet (UV) photons also damage amino acids and genetic material, while charged particles induce electroporation (41). A 5.3 log CFU/cm^2^ reduction of *Salmonella* on apple has been achieved after 240 s of DBD, and a 4.02 log CFU/mL *E. coli* reduction was demonstrated with apple juice via APPJ (39). In addition, cold plasma can improve nutrient retention and bioactive ingredient levels besides microbial inactivation. Iron bioavailability increases with plasma treatment due to surface activation and binding enhancement, also phytochemical concentrations increase due to membrane disruption, more than two folds increase in phenolics is reported for white grapes and 7.34% for blueberry juice (42). Vitamin C retention can reach 95% after 10 min might be due to NO-enhanced dehydroascorbate reductase activity (43).

Plasma assisted surface functionalization imparts oxygen and nitrogen functionalities for nutrient attachment. Ion bombardment and radial oxidation expand microporous structure, favorable for nutrient penetration, as rice fortification with effective iron binding (44). Safety assessment is still important for concern about remnant reaction species, antioxidant autoxidation and probable genotoxicity (38). Regulatory policies are in the process of being established, though a full toxicological evaluation and regulation as well as market and consumer acceptability criteria must be met before industry adoption (40).

### 2.3 Pulsed electric field

Pulsed electric field (PEF) is a novel non-thermal technology in terms of food fortification, utilizing short duration and high intensity electric pulses, leading to reversible or irreversible membrane electroporation to increase mass transfer out or into the cell for better nutrient bioavailability without bringing about adverse effect due to traditional thermal processing. Electric field strengths typically vary from 05 to 50 kV/cm, which are applied in microseconds pulses that in turn produce transmembrane potential differences above the dielectric breakdown threshold of cellular membranes (45). This electroporation effect creates nanopores in lipid bilayers that selectively permeabilize plant and microbial cells while maintaining bioactive compounds heat-sensitive (46).

Contrary to thermal fortification processes which can degrade thermosensitive vitamins, polyphenols and carotenoids, PEF allows with improving the extractability and bioavailability of these compounds with higher preservation yields (47). In food matrices, PEF impact is multi-dimensional, as it causes microstructural changes that facilitate subsequent processes (like drying and pressing for solid/liquid separation), maintains quality attributes such as color, flavor or texture. Fortification purposes show substantial enhancement of mineral, vitamin and phyto-chemical addition, improved extraction yield in anthocyanins, flavonoids and carotenoids from fruit and vegetable matrices There are many examples of such fortification applications, where substantial mineral vitamin and phytochemical incorporation improvements were observed, accompanied with increased extraction yields for anthocyanins flavonoids or carotenoids from fruit and vegetable matrices (48). The reversible electroporation at moderate field strengths allows controlled nutrient uptake, helping thus the rational nutrient fortification programs which tend to retain food nutritional and organoleptic characteristics (49). PEF-assisted extraction of nutraceuticals offers clear advantages over conventional solvent-based methods, including reduced extraction time, lower solvent and energy consumption, and comparable or superior yields, while preserving heat-sensitive bioactive compounds (50).

Electroporation is reversible and allowing the delivery of nutrients into cellular compartments with targeted delivery of fortified products which maintain nutritional and organoleptic proprieties (51). Applications also range from functional beverages and dairy products to plant-based food, in which PEF is beneficial for the integration of functional ingredients with no negative effect on stability or consumer acceptance. Recent progress has expanded the applications of PEF to strengthen emulsions, stabilize fortified emulsions, increase viability of probiotics and enhancing mineral bioavailability by modification of matrix (52). The flexibility of PEF parameters, e.g., pulse shape, frequency, and energy input permits optimization specific to matrices and fortificants being treated resulting in optimum nutrient retention with the desired functional changes (53).

### 2.4 Ultrasound processing

Ultrasound processing is an innovative non-thermal food fortification techniques providing process advantages over conventional thermal methods in the retention of biological activity and effectiveness of the incorporated fortificant. Its main mechanism is acoustic cavitation and it is based on the production and collapse of microbubbles in food matrix during successive compression (positive) and rarefaction (negative) cycles (51). In turn, these cavitation events generate shear forces, microjets, and shock waves breaking cell walls and establishing microchannels with enhances in porosity without involving extended periods of heat application. These structural changes improve mass transfer kinetics leading to a better dispersion and uptake of fortificants (50). However, its effectiveness is highly nutrient- and condition-dependent rather than universally applicable. Ultrasound-assisted fortification is particularly suitable for water-soluble vitamins (e.g., vitamin C and B-complex), minerals (iron and calcium), and polyphenolic compounds, where cavitation-induced microstructural disruption enhances diffusion, solubilization, and bioaccessibility (16).

Oxidative degradation may occur when high ultrasound intensity, prolonged treatment time, or oxygen-rich environments promote the formation of free radicals, particularly affecting oxidation-sensitive compounds such as polyunsaturated fatty acids, carotenoids, and fat-soluble vitamins (50). Therefore, process optimization using controlled intensity, pulsed operation, reduced exposure time, and inert or low-oxygen atmospheres is required to minimize oxidative losses (16).

From an industrial perspective, ultrasound offers advantages including relatively low capital investment, compatibility with liquid and semi-solid matrices, and ease of integration into existing processing lines. However, challenges related to scale-up, energy efficiency, cavitation uniformity, and equipment erosion at high power levels remain limiting factors for large-scale continuous processing (50). Compared with HPP, ultrasound exhibits lower microbial inactivation efficacy and reduced stabilization of lipid-soluble bioactives but offers greater flexibility for targeted fortification, shorter treatment times, and lower infrastructure requirements. Consequently, ultrasound is more suitable for small- to medium-scale or matrix-specific precision fortification applications, whereas HPP remains preferable for industrial-scale stabilization of sensitive bioactives and extended shelf-life products (29).

Iron content in the fortified yellow sweet potato increased from 2.11 mg/100 g in the control to 105.91 mg/100 g following ultrasound-assisted fortification conditions reported by Purizaca-Santisteban et al. (54), representing a 4,929% increase; ascorbic acid incorporation increased by 611% under the same study conditions. Approximately 18 times more calcium was present in the osmotically dehydrated apple slices compared with fresh apples. The highest mass fraction of B12 loading were successfully obtained from apple cubes (0.12–0.19 mg/g, dry basis) during optimized sonication (52), whereas iron-fortified pineapple chips enhanced with microencapsulation increased the iron content 1,000% and decreased drying time 35–52% reportedly (53).

The encapsulation efficiency (EE) of bioactives is also enhanced by ultrasound. Mulberry phenolics encapsulated in alginate were 73.88% efficient at recovering the ingredients, with retention levels of antioxidant activity ranging from 72 to 87% (55). The EE percentage of curcumin encapsulation in alginate–chitosan formulations was optimized at 9 min sonication (89.86%) due to the smaller particle size and stronger core–shell interactions, while excessive sonication led to recoalescence (56).

Ultrasound may also contribute to sustainable processing in terms of shorter processing time and energy conservation, but life-cycle assessments have been limited (57). Chlorogenic acid content was 2.47 times higher when mulberry leaves were optimally extracted by ultrasonication to give extracts of 168.17 mg GAE/g phenolics and antioxidant capacity of 1076.46 μmol TE/g (55). Acerola juice under high intensity ultrasound contributed to a higher value of B1, B3, B5, C vitamins, carotenoids and phenolics (58). The greatest benefit of ultrasound is the ability to control cavitation in a very precise way (with frequency, amplitude and duty cycle) to achieve an optimization in superimposition without causing degradation of nutrients.

The efficiency of ultrasound is its accurate control of the intensity of cavitation, by frequency, amplitude and duty cycle, to maintain elevated mass transfer while avoiding degradation of the nutrient (52). Ultrasound processing as non-thermal process is a pivotal technology for precision fortification program, to impart enhanced biological functionality with the advantages of retention in nutritional content, acceptable sensory qualities (59). Critical comparison of non-thermal technologies for food fortification summarized in Table 1.

**Table 1:** Critical comparison of non-thermal technologies for food fortification.

Technology	Best-suited fortificants	Main risk/limitation	Industrial readiness	References
High-pressure processing (HPP)	Fat-soluble vitamins, carotenoids, phenolics, minerals	High capital cost; limited suitability for dry foods	High (commercially established)	(11, 29, 34)
Ultrasound	Water-soluble vitamins, minerals, polyphenols	Oxidative degradation at high intensity; scale-up challenges	Moderate	(16, 50, 58)
Pulsed electric field (PEF)	Phytochemicals, intracellular minerals, juices	Limited effectiveness in solid foods	Moderate–High	(14, 45, 48)
Cold plasma	Surface mineral fortification, seed enrichment	Reactive species-induced oxidation; regulatory uncertainty	Low–Moderate	(38, 40, 42)
UV/pulsed light	Vitamin D precursors (ergosterol, 7-dehydrocholesterol)	Limited penetration depth	High (application-specific)	(61, 63, 64)
Supercritical CO₂	Lipophilic vitamins, carotenoids, omega-3 fatty acids	High operational cost; batch processing	Moderate	(62, 63, 72–76)

### 2.5 Ultraviolet light and pulsed light technologies ionizing irradiation technologies

Ultraviolet (UV) and pulsed light are two of the photochemical technologies used to strengthen foods antimicrobially, preserving foods by microbial decontamination without raising temperatures. UV-B irradiation (280–315 nm) leads to the photolytic cleavage of provitamin D precursors like ergosterol in mushrooms and 7-dehydrocholesterol, found in animal-based products, into previtamin D2 and D3 followed by isomerization to stable vitamin D types (60). Commercial mushroom fortification using controlled UV-B exposure (0.5–2.0 J/cm^2^) increases vitamin D2 to >1,000 IU/serving and does not alter sensory quality (61). Also, UV-C irradiation (254 nm) of bakery products after baking results in vitamin D2 addition; however, a precise dose is required to prevent off-odors and textural changes (62). Pulsed light systems delivering high intensity frequencies of broad-spectrum, e.g., 200–315 nm peak emission light in microsecond pulses offer processing efficiencies and thermal load reductions relative to continuous irradiation even when compared with vitamin D enhancement at matched fluence (63). Both UV and pulsed light lead to microbial inactivation by the formation of pyrimidine dimers and protein denaturation where reductions range from 1 to 4 log based on matrix and dose (64).

Ionizing irradiation processes, such as gamma rays (Cobalt-60, Cesium-137), e-beam and X-rays, penetrate deeper than UV radiation or heat and achieve complete microbial inactivation through ionization events that result induction of reactive oxygen species and free radicals leading to irreversible damage to cell membranes, proteins and DNA (65). Gamma irradiation is able to deliver the deepest penetration for bulk products, e-beam is designed for fast surface treatment, and X-rays provide non-radioisotope alternatives with comparable penetration capabilities (66). The stability of nutrients is related to dose, for example doses <5 kGy will generally have low effects on vitamins and minerals, while higher than 10 kGy can result in deterioration of thiamin, vitamin C, and vitamin E as well as oxidative changes in lipids and amino acids (67). Radiation sensitive products such as irradiation of vitamin D enriched dairy at 2–3 kGy prolongs shelf life with <10% loss in vitamin D along with good sensory quality (68). Consumer acceptance is main challenge, driven by misinformation about radioactivity and the negative connotations associated with it as irradiation (69). For which strategic communication highlighting safety, nutritional protection, and sustainability frequently with reference to cold pasteurization is increasingly successful at promoting acceptability (70).

### 2.6 Supercritical fluid extraction

Supercritical fluid extraction (SFE), especially with carbon dioxide (scSCo_2_) has been considered as a revolutionary with promising process for the recovery and fortification of bioactive compounds. CO_2_ is dense like a liquid but diffuses with the mobility of a gas, yielding an adjustable solvent platform that can tune selectivity by adjusting pressure and temperature above its critical point (31.1 °C, 7.38 MPa) (71). Such dual-phase behavior makes it possible to selectively extract specific compounds while maintaining thermally sensitive micronutrients, which is important for functional food applications (72). Addition of polar Co-solvents, like ethanol, increases the polarity range and allows the recovery of both lipophilic and medium polar bioactives. In contrast to the classical solvent extraction, scCO₂ works at low temperature, does not cause nutritional destruction and avoids toxic residue, conforming with a clean-label trend (62). scCO₂ works with absolute flexibility to phytochemical classes. Polyphenols and anthocyanins that are traditionally hard to extract intact can be selectively extracted with better antioxidant preservation than the traditional extraction methods (63). Carotenoids, specifically lycopene from tomato by-products, is recovered with high yields which are bioactive and further increased their oxidative stability through microencapsulation. Also, it is feasible to target enrichment of essential fatty acids such as omega-3 lipids using high-pressure-swing processes (73). Lipophilic vitamins, such as tocopherols and carotenoids are effectively extracted with minimal oxidation, whereas agroindustrial byproducts such as berry pomace and citrus peels are valorized into highly concentrated bioactive extracts for functional food uses (74).

Combining scCO_2_ with encapsulation methods is a revolutionary approach toward fortification. Emulsion-based methods allow direct preparation of food-grade micro- and nanoparticles with high encapsulation efficiency (73). Modern methods like particles from gas-saturated solutions (PGSS), rapid expansion of supercritical solutions (RESS), and supercritical antisolvent precipitation (SAS) allow the one-step extraction and encapsulation of bioactives into a matrix, thereby shielding bioactives from oxidation under storage conditions (75). These microencapsulated systems improve bioaccessibility and provide targeted release, which are critical components of personalized interventions (76). Lack of thermal stress also retain heat sensitive vitamins and polyphenols, and the use of scCO₂ extracted ingredients is in line with clean label developments based on consumer demand for natural, less processed functional foods (77). The application of non-thermal technology in food fortification are summarized in Table 2.

**Table 2:** Non-thermal technology application in food fortification.

Non-thermal technology	Processing parameters	Food matrix/substrate	Primary applications	Key mechanisms	References
Pulsed electric field (PEF)	Electric field strength: variable; Frequency: variable; Pulse width: variable; Treatment time: variable; Specific energy input: variable; Short-duration, high-voltage pulses	Fruit juices, vegetable juices, milk, plant tissues, plant-based beverages	Microbial inactivation; Enzyme inactivation; Extraction of bioactive compounds; Preservation; Membrane permeabilization	Cell membrane electroporation; Increased membrane permeability; Enhanced compound extractability; Minimal heat generation	(175)
High pressure processing (HPP)	Pressure: 450–600 MPa; Treatment time: 3 min (typical); Temperature: ambient to moderate	Fruit smoothies, liquid foods, plant-based beverages, diverse food matrices	Preservation; Shelf-life extension; Extraction of bioactive compounds; Microbial inactivation; Enzyme deactivation	Cell permeabilization; Structural changes in food matrix; Enhanced extractability; Prevents denaturation of bioactives	(31)
Ultrasound (US)	Power: 183–373 W/cm^2^; Frequency: variable; Treatment time: 10 min (typical); Intensity: variable; Amplitude: variable; Nominal power: variable	Fruit juices, plant-based beverages, plant proteins, vegetable-based products, dairy alternatives	Microbial inactivation; Extraction enhancement; Functionalization; Improving protein solubility; Enhancing bioaccessibility	Cell wall disruption; Cavitation effects; Enhanced mass transfer; Improved compound release; Microstructural modifications	(189)
Cold plasma (CP)	Glow discharge plasma: Air flow: 10–30 mL/min; Dielectric barrier discharge (DBD): Frequency: 50-1000 Hz; Treatment time: 20 min	Fruit juices (orange, cashew apple), fermented foods, fresh produce	Microbial decontamination; Surface sterilization; Bioactive compound enhancement; Preservation	Reactive oxygen species (ROS) generation; Surface-selective oxidative chemistry; Membrane permeability changes; Biosynthesis induction	(178)
Pulsed light (PL)	Light intensity: variable; Wavelength range: UV to visible; Pulse duration: short; Treatment time: variable	Plant food matrices, fresh produce	Surface decontamination; Microbial inactivation; Bioactive compound enhancement	Photochemical effects; Biosynthesis induction; Minimal membrane permeabilization	(190)
UV treatment	UV wavelength: variable; Intensity: variable; Exposure time: variable	Fresh produce, liquid foods	Surface decontamination; Microbial inactivation; Preservation	DNA damage in microorganisms; Photochemical reactions	(191)
High pressure homogenization (HPH)	Pressure: high; Flow rate: variable; Number of passes: variable	Plant-based beverages, vegetable tissues	Particle size reduction; Emulsion stabilization; Enhanced extractability; Improved bioaccessibility	Mechanical disruption; Microstructural changes; Enhanced compound release	(192)
Ozone treatment	Ozone concentration: variable; Contact time: variable; Temperature: ambient	Fresh produce, liquid foods	Decontamination; Pesticide residue reduction; Microbial inactivation	Oxidative reactions; Cell membrane disruption	(190)
High-pressure carbon dioxide	Pressure: high; CO₂ concentration: variable; Temperature: moderate; Treatment time: variable	Liquid foods	Microbial inactivation; Enzyme inactivation; Preservation	pH reduction; Cell membrane disruption; Enzyme inactivation	(191)
Radiation processing	Dose: variable; Type: ionizing radiation; Exposure time: variable	Liquid foods, fresh produce	Sterilization; Preservation; Shelf-life extension	DNA damage; Microbial inactivation; Enzyme inactivation	(60)
Oscillating magnetic field	Magnetic field strength: variable; Oscillation frequency: variable; Treatment time: variable	Diverse food products	Microbial inactivation; Nutrient preservation	Electromagnetic disruption	(193)

## 3 Omics technologies in nutritional sciences

### 3.1 Genomics and nutrigenomics

The integration of genomics and nutrigenomics has revolutionized nutritional sciences for personalized nutrition interventions. Nutrigenomics investigates the interaction between nutrients and genes whereas genomics identifies genetic variations influencing nutrient metabolism, absorption, and requirements (78). Enzymes and transporters are markedly influenced by Single Nucleotide Polymorphisms (SNPs) in nutrient metabolism (79). For instance, BCO1 variants (rs6564851-C and rs6420424-A) exert effects through circulating lutein and zeaxanthin on carotenoid metabolism and vitamin A status, while CETP rs708272 modulates high-density lipoprotein (HDL)-cholesterol response to diet, suggesting the roles of genetic architecture in association with lipid metabolism (18).

Gene-diet interactions are a focus of nutrigenomics research as differing diet patterns will cause different effects in relationship to genetic background (80, 81). Mediterranean diets modulate insulin resistance in carriers of 9p21 risk alleles (82), and risk allele variants at ZPR1, BUD13, and ALDH1A2 interact with energy consumption to modify HDL-cholesterol (78). Genetic susceptibility for nutrient deficiencies is due to defects in absorption, conversion or transport. Variants have been associated with modified levels of vitamin D, folate, iron, calcium, vitamin A and omega 3, which may be informative toward genotype-guided supplementation strategies (83). Epigenetic changes also play a role in nutrition-gene interactions, various nutrients have been shown to be associated with levels of DNA methylation, histone modification, and noncoding ribonucleic acid (RNA) expression which results in alterations in metabolic pathways and disease risk of phenotypes (84). Integrating epigenomics with transcriptomics, proteomics and metabolite profiling enables comprehensive mapping of nutrient-induced molecular alterations (78). The potential of precision nutrition relies on rigorous validation, open access to genetic testing and evidence-based algorithms that back translation from genomic insights to personalized dietary advice based on the unique genotype of an individual (79).

### 3.2 Transcriptomics

Gene expression profiling under controlled nutrient interventions is unveiling discrete transcriptional programs that connect dietary components with metabolic outcomes, establishing mechanistic layout for personalized nutrition. Omics-based profiling enables subgrouping of people based on molecular response signatures, directing personalized dietary recommendation (85). Transcriptomic approaches provide mechanistic insight into how fortified foods and processing technologies modulate biological responses at the gene expression level. In contrast to general dietary exposure studies, transcriptomic investigations of fortified foods focus on nutrient-specific signaling pathways, transporter regulation, and cellular stress responses associated with enhanced micronutrient delivery (86). Importantly, non-thermal processing technologies can modulate transcriptomic responses indirectly by preserving bioactive integrity and altering food matrix interactions. HPP-treated fortified foods have been shown to enhance cellular antioxidant response pathways and nutrient-responsive gene networks compared with thermally processed counterparts, reflecting improved bioaccessibility and reduced processing-induced stress signals (87). Similarly, PEF processing has been associated with altered expression of genes related to membrane transport, mineral uptake, and oxidative stress regulation in intestinal and epithelial cell models exposed to fortified matrices (88).

The transcriptomic signatures provide functional validation that non-thermal fortification strategies influence not only nutrient retention but also downstream biological responses relevant to precision nutrition. Consequently, transcriptomics serves as a critical bridge between food processing technology and personalized health outcomes, enabling the identification of nutrient-responsive biomarkers that can guide fortificant selection, processing conditions, and dose optimization in omics-informed fortification frameworks (89).

Recently, RNA sequencing (RNA-seq) has emerged as the most powerful medium to capture diet-related alterations in both coding and noncoding transcripts, allowing for the discovery of biomarkers and their further development into a quantitative one. RNA-seq helps to dissect the molecular basis for reprogramming nutrient-sensing and aging-related pathways by calorie restriction, macronutrient modulation, and other interventions in humans and model organisms (90). For instance, in kitten feeding trials it was demonstrated that supplementation with nucleotides and uligosaccharides resulted in decreases of circulating miR-1-3p and related species (91). MicroRNAs (miRNAs) are increasingly recognized as key post-transcriptional effectors of nutrients. Effects of early-life macronutrient exposure on hundreds of hypothalamic miRNAs are involved in pubertal timing and endocrine programming (92). Integrated miRnomics and mRNA expression profiling predicts the potential regulatory pathway and downstream targets in obesity-related liver disease (93).

Transcriptomics analysis further illustrates that a nutrient response is very tissue-specific. Comparative RNA-seq between the brain, liver, adipose tissue and muscle reveals different gene and isoform responses to identical interventions that have large effects in some tissues but only small or no changes in others tissue (94). Similar dietary effects on the liver metabolism, muscle isoform usage and immune transcription programs have been shown in other livestock and fish. Chromatin-centric studies demonstrate that vitamins such as vitamin D regulate tissue-specific chromatin and transcriptional dynamics with implications for immune and muscle function (95). These observations highlight the need for tissue-informed transcriptomic profiling in precision nutrition and development of non-thermal fortification strategies specific to desired molecular outcomes.

### 3.3 Proteomics

Proteomics has become an invaluable tool in nutritional sciences by facilitating the broad characterization of protein expression dynamics, post-translational modifications (PTMs), and functional protein–protein interactions upon dietary interventions. Progress in mass spectrometry and high-throughput affinity assays have made it possible to quantify thousands of proteins simultaneously, revealing new insights into diet–health interrelations (96). They allow for the discovery of biomarkers associated with nutritional status and prediction of dietary response, and contribute to the mechanistic elucidation of nutrient-protein interactions that underlie metabolic health (97).

Dietary interventions result in rapid and persistent alterations of plasma and tissue proteomes, some proteins acting as sensitive reporters of metabolic adaptation. Both targeted affinity panels and quantitative proteomics indicate that fibroblast growth factor 21 (FGF-21) is predictive weight-loss success while apolipoprotein C1 (APOC1) is responsive acutely to caloric deprivation and a glucose challenge (98). High throughput profiling studies show changes in sex hormone-binding globulin (SHBG), adiponectin, C-reactive protein (CRP), calprotectin, serum amyloid A, and proteoglycan 4 (PRG4) that correlate with insulin sensitivity and inflammation status (99). Unique proteomic profiles in diet-resistant obesity, such as increases of heat shock protein 72 (HSP72) and eukaryotic translation initiation factor 5 (eIF5), indicate an inherent heterogeneity for stress responses and the cellular machinery that synthesize proteins (100).

PTMs provide a vital regulatory level in which diet controls protein function. Phosphorylation and N-glycosylation of mitochondrial and metabolic proteins are reprogrammed by obesogenic diets that impact phosphoglucomutase 1 (PGM1) and monocarboxylate transporter 1 (MCT1). These changes link nutrient exposure to mitochondrial-associated dysfunction and the risk of metabolic diseases using site-specific enrichment approaches for PTM that promote specific mechanistic insights (101). Proteomic biomarkers are a very promising way to approach personalized nutrition, as they allow to stratify the subjects based on the predicted response to the diet and to follow the effectiveness of the intervention (102). An index of markers consisting of inflammatory plus metabolic protein groupings predicts body mass index trajectories and follows longitudinal changes during weight loss interventions (99). Proteomics is also helpful in the analysis of allergen presence in complex food matrices and functional protein-nutrient interactions, exosomal proteins, that improves myotube respiratory capacity in diet-sensitive subjects. Integration with other omics analysis will yield information on the molecular signatures underlying future evidence-based personalized nutrition interventions (102).

### 3.4 Metabolomics

Metabolomics has evolved as a powerful analytical methodology in the area of nutritional sciences for profiling small molecule metabolites in biological systems, and provides unprecedented opportunities for studying rapid responses to dietary interventions. This omics assay, allows the estimation of the downstream consequences of changes in genomic, transcriptomic and proteomic levels which translates through functional readouts about physiological conditions that are directly related to individual reactions toward non-thermal approaches aimed at food fortifications. The use of metabolomics in personalized nutrition has led to a significant improvement in ability to measure nutritional status, identify biomarkers linked to dietary intake and investigate metabolic phenotypes responsible for inter-individual variability in nutrient metabolism and associated health (103).

Metabolic phenotyping Metabolic phenotyping is a cornerstone application of metabolomics in the nutrition assessment, and researchers use it to categorize individuals according to their the metabolic profile of an individual and that individual’s potential response to specific dietary intervention (104). Current state-of-the-art analytical platforms, e.g., nuclear magnetic resonance spectroscopy and mass spectrometry in combination with chromatographic separation techniques, allow simultaneous identification and quantification of several hundreds to thousands of metabolites within biological samples such as plasma, urine or feces (105). These complex metabolic phenotypes capture the interaction between genetic predisposition, gut microbiome composition and dietary style as well as environmental lifestyle influences, resulting in a whole-body perspective of one’s nutritional and health status. The combination of metabolomics with other omics techniques has been particularly successful in the discovery of novel dietary intake biomarkers, providing information beyond traditional self-reported assessments on the actual food intake and nutrient bioavailability (106). Lipidomics allow the profiling and identification of lipids in a complex biological sample and has brought insight into this inter-individual variability, especially concerning dietary fat composition, very little is known on how certain diets confer protection against CVD and metabolic syndrome. Currently, the targeted and untargeted lipidomics has provided a comprehensive description of fatty acid profiles, phospholipid species and bioactive lipid mediators to control lipid metabolism during personalized dietary fat recommendations, depending on different metabolic phenotypes (107).

### 3.5 Microbiomics

The development of multi-omics techniques has revolutionized nutrition research and enabled to better understand the contribution of gut microbiome to nutrient metabolism and personalized dietary treatment approaches. Metagenomic sequencing, metabolomics, and metatranscriptomics studies have identified intricate links between intestinal microbiota composition, dietary behaviors and host metabolic responses supporting the emerging concept of precision nutrition designed to meet specific microbiome profiles (108). Gut microbiomic health status mainly depends on its composition and diversity. In a large study of deep metagenomic sequencing from >1,000 participants, strong correlations were observed between alpha diversity, species richness and habitual diet with metabolic traits with consistent associations across populations (109). Personalized interventions based on microbiome profiling are effective in the clinic while randomized study showed increased Shannon diversity, improved glycemic control (HbA1c decreased from 8.30 ± 1.12 to 6.67 ± 0.89, *p* < 0,001) and a negative delta of CRP of 19,5%, highlighting potential for microbiome stratification (110).

The nutrition metabolism via the microbiome is an important interface between diet and host physiology. In this context, short-chain fatty acids (SCFAs), especially butyrate, have been proposed as the driving force of host–microbiome crosstalk. Personalized responses to prebiotics as a means of intervention are shown (pseudo-*R*^2^ = 0.39) that surpass the prebiotic itself-dependent effects (pseudo-*R*^2^ = 0.05), then evidencing individuality in metabolic outcome (111). Integration of multi-omics also reveals that the microbiome predicts postprandial lipemia better than glycemia, demonstrating its complex set of regulatory levels (109).

Probiotics and prebiotics in fortified foods have a specific modulated microbiome. Infant formulas supplemented with pre- and postbiotics have exhibited fecal microbiota and metabolites that are similar to the breastfeeding infant, with 404 differing as compared to controls (112). Machine learning-driven omics analyses unveil mechanisms of prebiotic efficacy showing that there is an increased SCFA production and a beneficial modulation of taxa such as *Bifidobacterium* and *Faecalibacterium* (113). The host-microbiome-nutrient interface is remarkably individualized that depends on host baseline microbiome composition, habitual fiber intake, and SCFA concentrations (114). Novel paradigms that integrate metagenomics, metabolomics and host transcriptomics computational frameworks with network analysis and artificial intelligence will provide precision nutrition approaches that tailor the microbiome composition for optimally health of both the microbes and their host (115).

### 3.6 Multi-omics integration

**Figure 2:**
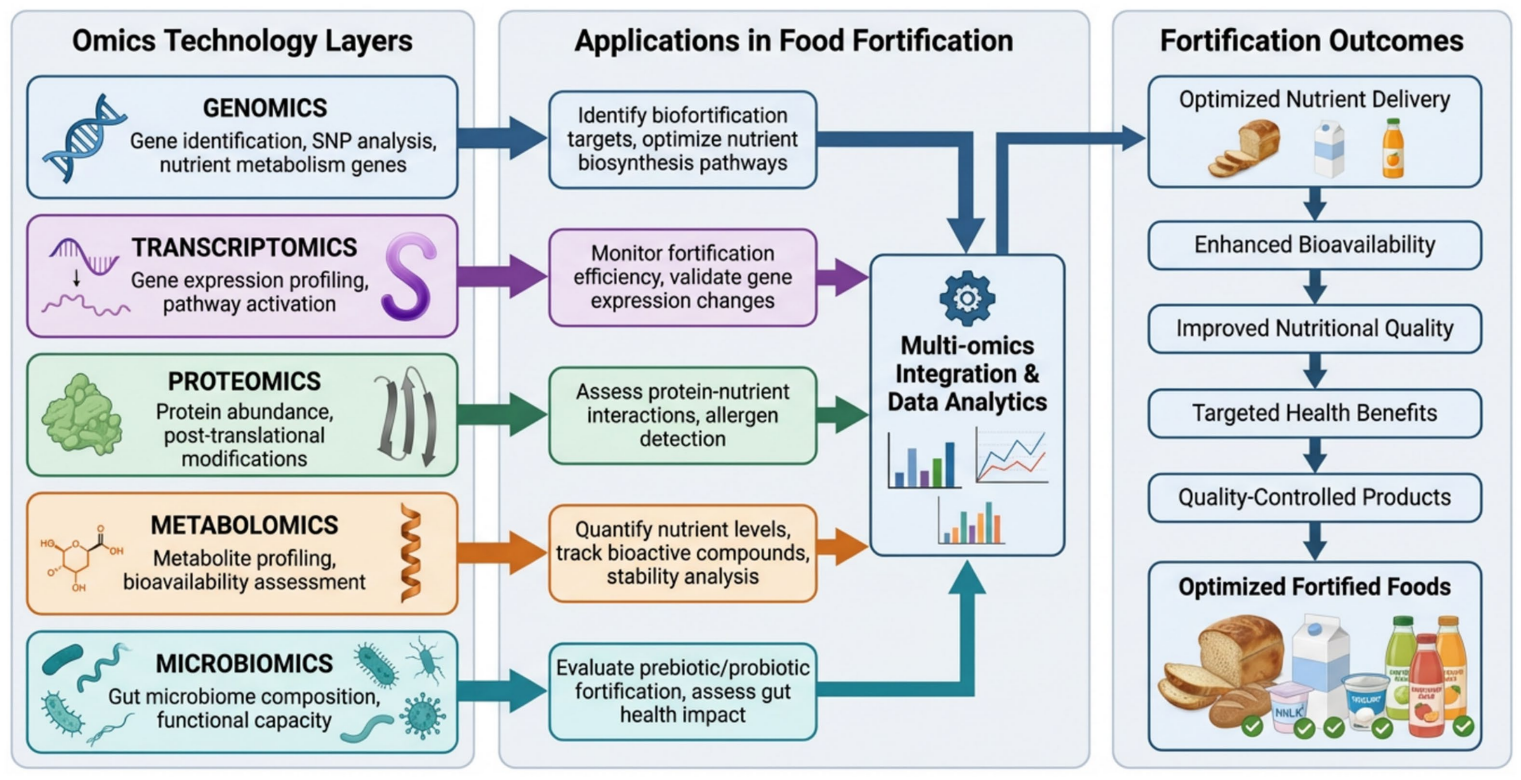
Multi-omics approaches for optimizing food fortification strategies.

The integrated use of omics technology in nutritional sciences enables worldwide understanding of the intricate biological system of interaction between individual reactions to dietary interventions and non-thermal fortification plans shown in Figure 2 (116). System biology approaches combine data of the genome, transcriptome, proteomics, metabolome and microbiome to form systems wide models that capture the dynamic response between a genetic predisposition environment and nutritional condition. Such integration provides distinctive opportunities of personalized nutrition (117). Technological progress in computational infrastructure and bioinformatics technologies that allow pooling disparate data types, correcting technical variation, and extracting meaning out of high-dimensional data sets (118). Networks and pathways Network analysis and pathway mapping are core in multi-omics integration where graph-theoretical based methods applied to find regulatory hubs, metabolic bottlenecks and coordinated modules in response to diet challenges. Such methods provide data regarding the consequences of genetic variations on protein expression and metabolite flux via specified pathways (119). Machine learning approaches have been increasingly applied to integrate multi-omics datasets in nutrition research, enabling the identification of diet–response phenotypes and prediction of individual variability in nutrient metabolism. Supervised learning models, including random forests and gradient boosting algorithms, have been successfully used to integrate genomics, metabolomics, and microbiome data to predict postprandial glycemic and lipemic responses in human dietary intervention studies, demonstrating practical utility beyond theoretical modeling (120).

AI-driven frameworks have been employed to link omics-derived biomarkers with processing conditions, where multivariate learning models associate nutrient retention and bioaccessibility outcomes with non-thermal processing parameters such as pressure intensity, electric field strength, and treatment duration. Although applications directly combining non-thermal fortification and AI-guided omics optimization remain limited, these approaches provide validated foundations for translating multi-omics data into predictive decision-support tools for fortification design (85). Future progress will depend on well-annotated datasets, standardized omics pipelines, and experimental validation linking AI predictions to measurable fortification and health outcomes (117).

## 4 Food fortification strategies using non-thermal technologies

Food fortification is a core public health strategy to tackle micronutrient deficiencies and improve food and nutrition status in communities, with non-thermal processing technologies providing guidelines for preservation of nutrient bioactivity and improvement in bioavailability (5). Strategies for fortification fall into one of two categories. Biofortification, the introduction of higher levels of nutrients during crop maturation using agronomic or genetic methods, and industrial fortification, the addition of nutrients during food processing (4). Target nutrients are selected based on evidence of local deficiencies from epidemiological data, and vehicle selection is predicated on the potential for high consumption levels, low cost, ease of central processing, and lack of sensory issues. The fortificant matrix has to offer a trade-off between stability, solubility, sensory effects and cost-effectiveness with non-thermal processes being superior in preservation of heat-labile compounds (121).

Micronutrients fortification includes all the vitamins and minerals. Fat-soluble (vitamins A, D, E and K) require lipids compatible matrices for their delivery, whereas water- soluble vitamins (B complex and C) are susceptible to oxidative degradation and thermal instability which can be overcome using non-thermal strategies as well (1). Mineral fortification with iron, zinc, calcium, iodine and selenium require consideration for relations to the matrix, formation of insoluble complexes and competitive absorption as well as deleterious pro-oxidant effects (122). The addition of trace elements requires high accuracy in dosing to achieve levels where efficacy is found and toxicity is prevented, with protective technologies avoiding oxidation, chelation, and precipitation. Omics technologies will permit customized evaluation of micronutrient needs and absorption capacity so that fortification strategies can be tailored for specific, genetically determined metabolic polymorphisms (123).

Fortification is also becoming more widespread for bioactive compounds and those with health functions (114). Although polyphenol and antioxidant compounds are not stable or well bioavailable so their fortification issues are addressed using non-heating encapsulation systems (124). Omega-3 fatty acids such as eicosapentaenoic acid (EPA) and docosahexaenoic acid (DHA) are very sensitive to oxidation; thus microencapsulation and co-fortification with antioxidants are required (125). Phytosterols and carotenoids are dependent on specific delivery systems, emulsion-based technologies and lipid nanoparticles improve solubilization and absorption (126). The addition of probiotic and prebiotic is complex to be maintained in terms of microbial viability and functionality, with the thermal technologies being quite harmful as they are heat sensitives (127, 128).

Fortifying with protein and amino acids (AAs) counter proteins energy malnutrition. Diet supplementation with limiting amino acids such as lysine and methionine enhances the protein quality of cereal-based diets (128). Fortification of plant-based protein using legumes, oilseeds and novel sources in isolates or concentrates help fill the nutritional gap in vegetarian and vegan diets, while supporting toward sustainability (129). In addition to composition, the quality of a protein depends on its digestibility and allergenicity. Non-thermal processing could modify protein structure and improve digestibility, reduce allergenic epitope in Soybean (130). New encapsulation materials and processes have transformed fortification by protecting vulnerable nutrients and allowing for the delivery of active ingredients to specific sites in the gastrointestinal (GI) tract. Nano-encapsulation strategies have been developed for nanoliposomes, polymeric nanoparticles and nanoemulsions to improve bioavailability by augmenting surface area for epithelial contact (131). Non-thermal-based microencapsulation techniques, including spray drying, freeze-drying, and coacervation, as well as ionic gelation are attractive due to their efficiency of encapsulation, release kinetics and scalability (132). These wall materials, such as proteins, polysaccharides and lipids help nutrients by masking them from oxygen, light, moisture and extremes of pH to prevent oxidation by preventing such exposure while the mechanisms for controlled release are optimized so that efficient absorption takes place at proper timing meanwhile reducing losses (133).

Nanotechnology and materials science have further developed advanced carriers for bioactives. Liposomes and niosomes, which are good with biocompatibility and possess a property of fusion with the membrane, encapsulate hydrophilic as well as lipophilic nutrients (134). Solid lipid nanoparticles are considered the first generation lipid-based carrier system allowing controlled release of lipids and also protection against degradation, while still being GRAS (Generally Recognized As Safe) status. The systems like nanoemulsions or multiple emulsions that are based on emulsion deliver lipophilic bioactives in improved stability and augmented bioavailability by taking advantage of fine droplet sizes and interfacial engineering (135). Novel smart delivery systems comprising stimuli-responsive polymers and targeted ligands are the future of tailored nutrition allowing nutrients to be released in response to physiological parameters and tissue-directed targeting based on omics-informed markers (136).

## 5 Integration of omics and non-thermal fortification

### 5.1 Omics-guided fortification design

The integration of omics tools toward food fortification signifies a paradigm shift from broad-based supplementation to precision nutrition customized to population requirements and individual genetic susceptibilities. Genomics techniques used for the study of nutrient requirements through the discovery of polymorphisms that have an impact on metabolism, absorption and utilization (23). Polymorphisms in metabolic enzymes, transporters and receptors could also significantly modify responses to fortified foods which would require tailored strategies (18). For example, polymorphisms of the MTHFR gene influence folate metabolism and have driven efforts at population based folate fortification in populations with a high allele frequency (137). Also, vitamin D metabolism and iron homeostasis-associated variants have been revealed through Genome-Wide Association Study (GWAS), which provides evidence for population-specific strategies. Integration of pharmacogenomics and nutrigenomics by applying the principles of pharmacogenomics to nutrigenomics allows for prediction of individual responses, moving toward personalized fortification strategies that provide for maximizing benefits without exceeding safe doses (138) (Table 3).

**Table 3:** Integrated multi-omics and non-thermal processing technologies for food fortification.

Omics technology	Non-thermal technology	Food fortified application	Key findings	References
Metabolomics and transcriptomics	Cold plasma	Broccoli sprouts	Cold plasma treatment (2 min) significantly enhanced sulforaphane, glucosinolates, total phenols, and flavonoids content, along with increased myrosinase activity. Transcriptomic analysis revealed upregulation of genes encoding biosynthesis pathways for these bioactive compounds.	(194)
Untargeted metabolomics	Ultrasound (Ultrasonication)	Soybean okara protein fortification	Ultrasound-assisted enzymatic hydrolysis produced bioactive peptides with enhanced antioxidant properties, DPP-IV inhibition (39.54%), and ACE inhibition (30.54%). Improved bioavailability and safety profiles of peptides for functional food applications.	(195)
Metabolomics	Freeze-drying (Non-thermal preservation)	Pumpkin flower powder for food fortification	Metabolomics profiling identified bioactive compounds and techno-functional properties. Enhanced understanding of nutritional composition for developing functional foods with specific dietary requirements.	(196)
Metabolomics	Pulsed electric field (PEF)	Carrot and tomato products	PEF pretreatment significantly enhanced bioaccessibility of carotenoids (*β*-carotene, lycopene) and phenolic compounds. Metabolomics profiling confirmed improved nutrient retention and bioactive compound stability.	(197)
Metabolomics	HPP + PEF	Rosehip infusion	Combined metabolomics analysis of HPP and PEF-treated rosehip infusions showed superior phenolic bioavailability compared to thermal and commercial pasteurization. Non-thermal methods preserved anthocyanins and vitamin C more effectively.	(198)
Metabolomics + Proteomics	Cold plasma	Chicken breast powder	Cold plasma treatment induced multifaceted changes in physicochemical and nutritional attributes. Metabolomics and proteomics revealed protein oxidation patterns, lipid modifications, and formation of bioactive peptides with enhanced functional properties.	(199)
Proteomics	HPP + PEF + Ultrasound	Dairy products	Proteomic analysis of dairy products processed with innovative non-thermal technologies revealed protein structural changes, improved digestibility, and enhanced bioactive peptide release. Maintained nutritional quality while ensuring safety.	(200)
Metabolomics	UV + Irradiation	Fruit and vegetable products	Metabolomics profiling showed non-thermal irradiation preserved heat-sensitive nutrients while enhancing bioactive compound extractability. Improved carotenoid and polyphenol bioaccessibility in processed products.	(192)
Proteomics + Metabolomics	PEF	Milk and whey products	Comparative proteomics and metabolomics analysis demonstrated PEF maintained superior nutritional attributes compared to traditional pasteurization. Enhanced retention of immunoglobulins, lactoferrin, and bioactive peptides with reduced bacterial load.	(201)
Metabolomics + Lipidomics	Ultrasound + HPP	Fortified beverages	Combined metabolomics and lipidomics showed ultrasound and HPP improved emulsion stability and nutrient encapsulation in fortified beverages. Enhanced bioavailability of fat-soluble vitamins (A, D, E, K) and omega-3 fatty acids.	(202)
Metabolomics	Supercritical CO₂ (Non-thermal extraction)	Bioactive compound extraction	Metabolomics profiling of supercritical CO₂ extracts showed superior recovery of thermolabile bioactive compounds. Enhanced bioavailability of polyphenols, carotenoids, and essential oils for functional food fortification.	(192)

Metabolomics used to expand the information generated in genomics by evaluating fortification efficacy from a perspective of global metabolic profiling. Untargeted approaches reveal new biomarkers and unsuspected metabolic outcomes (139), while targeted metabolomics quantifies nutrients and metabolites to follow bioavailability and nutritional fate. Integration with clinical end-points shows that folic acid fortification increases the concentration of circulating nutrients and alters downstream pathways related to health promotional effects (19). Longitudinal metabolomics can be used to discover adaptive responses and determine optimal dosing regimens, as well as synergistic or antagonistic interactions between fortified nutrients and food components to maximize matrices (140).

Mechanistic insights into nutrient uptake and utilization are derived from proteomics. Intestinal cell line quantitative proteomics studies reveal how fortification regulates transporter expression and inform selection of chemical form and delivery system (141). Phosphoproteomics connects signaling cascades to physiological effects, and protein-nutrient interaction studies illustrate the impact of response factors in food matrices on bioavailability (142). Saliva and plasma proteomics have identified fortification-sensitive biomarkers that represent noninvasive monitoring markers (143).

**Figure 3:**
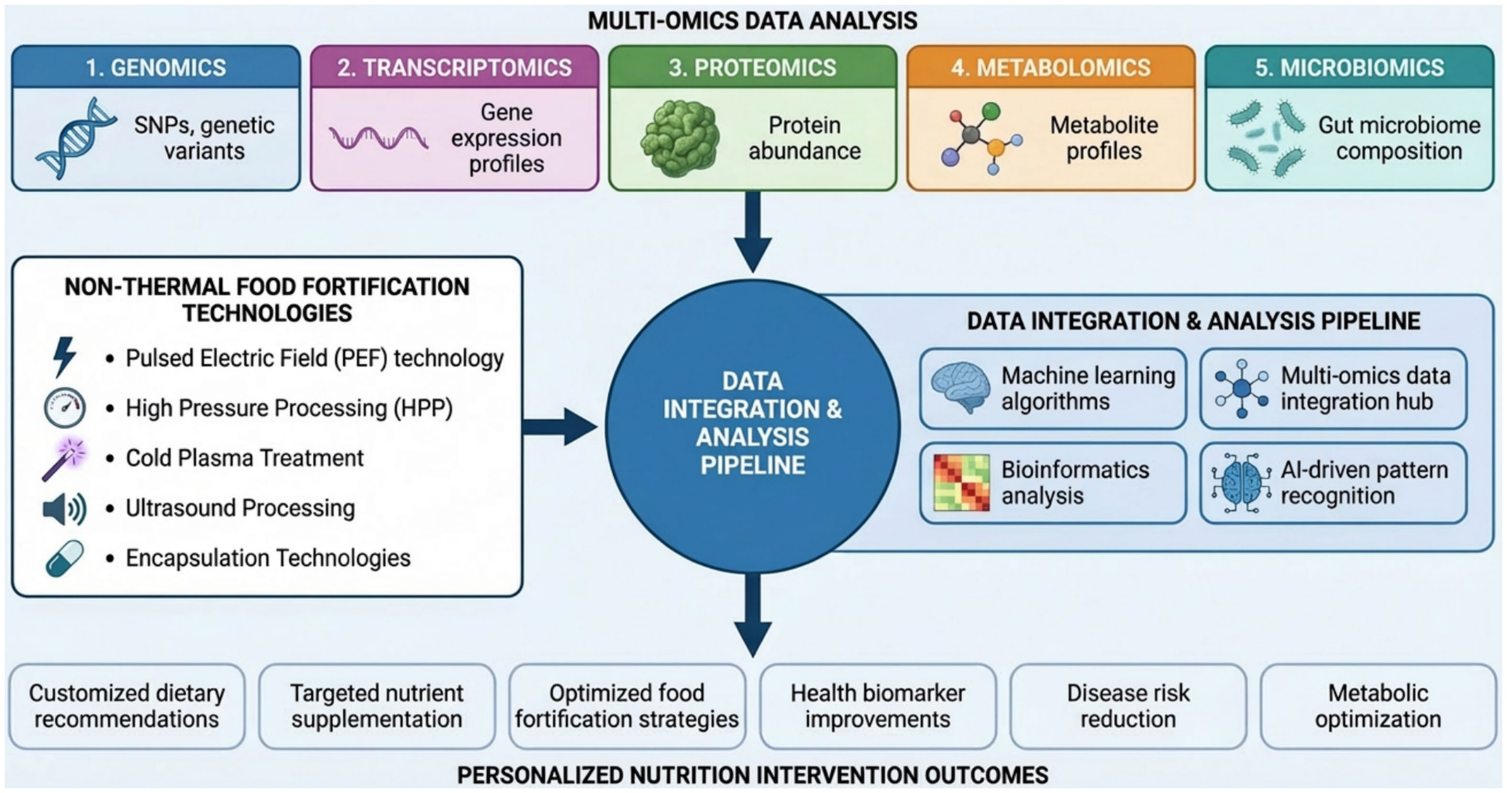
Integrated multi-omics and non-thermal processing technologies for personalized nutrition.

Microbiomics allow to study the relationship between gut microbes and nutrient metabolism. Metagenomics of the gut had shown that there are population-specific microbial capabilities to synthesize vitamins and produce SCFAs (144). Metatranscriptomics and metaproteomics studies showed that probiotic fortification promotes nutrient bioaccessibility (145). Such strain-specific genomics can be used to select appropriate probiotic strains possessing desired metabolic activities. The synergestic strategies involving prebiotics, probiotics and nutrients offering personalized strategies that converge with microbiome profiles (146). The schematic view of integration of multi-omics techniques with non-thermal processing technologies for personalized nutrition shown in Figure 3.

### 5.2 Precision fortification approaches

Precision fortification is a targeted interventions approach that exploit omics technologies to match the selection and dose of fortificants to specific biological phenotypes and metabolic requirements (23). Advances in genomics make possible genotype-specific strategies based on polymorphisms that influence nutrient metabolism, absorption, and availability. Notably, MTHFR polymorphisms that influence folate demand, FUT2 genotypes affect the vitamin B12 status (147). Using the principles of pharmacogenomics to nutrition, nutrigenomics focuses on genotype-specific fortification advice to maximize absorption and avoid harmful reactions by SNPs in transporters, enzymes, or receptors as measurable endpoints (18). Genome-wide associated polygenic risk scores have also emerged that predict susceptibility to nutrient deficiencies, and chronic diseases and thus enable preliminary fortification strategies for managing genetic predispositions before clinical presentation (148).

The age-specific fortification programs also address changing nutritional requirements during different stages of life. Pediatric fortification focuses on iron, zinc, vitamin D and omega-3 fatty acids for neurodevelopmental processes, skeletal growth and immune maturation (149). Adolescents need appropriate interventions considering their rapid growth and changes in hormones, which primarily focuses on iron for menstruating females and protein for muscle development in males. Geriatric fortification replaces diminished stomach acid, intrinsic factor, and absorptive capacity with high doses of vitamin B12, calcium, vitamin D, and protein to avert sarcopenia and bone density preservation (150). Sex-specific needs go beyond reproductive health and into lifestyles; for example, women require as iron fortification to counter iron losses from menstruation and men also benefit from selenium and lycopene in the prevention of prostate disorders (151).

Precision fortification is also influenced by ethnicity and geography. Lactase persistence polymorphisms are found at varying frequencies in populations and affect the appropriateness of using dairy-based products, as well as a need for alternatives for lactose-intolerant groups in certain communities such as East Asian, African, and Native American populations (152). Disease-specific approaches will also apply to individuals with a high-risk profile identified through the omics biomarkers, such as antioxidant fortification to prevent cardiovascular disease (CVD), omega-3 enrichment for inflammatory conditions, and prebiotic fortification for gut dysbiosis (153). An integration of multi-omics profiling, non-thermal technologies and precision nutrition principles will enable truly personalized nutrition solutions that maximize health benefits, while ensuring respect for bioindividuality, cultural preference and sustainability.

### 5.3 Quality assessment using omics

Multi-omics methodologies have recently revolutionized the quality control of fortified foods allowing a complete assessment of such factors as nutritional enrichment, processing effects and product authenticity. Critically, metabolomic profiling allows for an extensive description of biochemical composition, encompassing endogenous and exogenous metabolites brought in as part of fortification (154). Mass spectrometry instruments can measure hundreds of bioactives compounds such as vitamins, minerals, polyphenols and phytonutrients, which verify fortification levels as well as identify degradation products. Nuclear magnetic resonance (NMR) techniques allowed for monitoring structural integrity and chemical stability during non-thermal processing (155). Metabolomic fingerprinting can differentiate fortification processes and estimate nutrient bioavailability in complex food matrices (19). Proteomics provides a wealth of information on protein structure, activity, and protein–nutrient complex formation during non-thermal processing. High-resolution proteomics identifies modifications in nutrient binding and digestibility induced by HPP and PEF treatment in porcine musculature, which are involved in the nutrition-retaining effect of HPP and PEF processed meat products (156). Proteomic allergen analysis is of critical importance for consumer safety, particularly for novel protein sources (157).

Transcriptomics offers a mechanistic assessment of biological action. Gene expression profiling in cell and animal models demonstrates that fortified foods influence metabolic pathways, inflammatory responses, and oxidative stress (158). RNA-sequencing, mapping the transcriptional changes induced by bioactives and microRNA profiling as sensitive markers for personalized nutrition. Newly developed single-cell transcriptomics demonstrates diverse cellular responses, leading to a deeper understanding of the variability among individuals (18).

Integrity and traceability remain crucial in the fortified food value chain. Metabolomic and proteomic fingerprinting of geographical origin, processing history and authenticity (159). Isotope ratio analysis coupled with metabolomics confirms nutrient sources, and genomic techniques such as DNA barcoding and whole-genome sequencing provide identification of botanical/animal input in a highly accurate manner (160). Integrating the multi-omics data with chemometrics and machine learning methods increase the authenticity level. Blockchain technologies integrated with omics-based help for authentication, transparency and traceability of fortified food systems (161).

### 5.4 Bioavailability and bioaccessibility studies

The bioavailability and bioaccessibility of nutrients in fortified foods are very fundamental in the efforts to ensure nutritional intervention yields desired health outcomes especially when non-thermal processing methods are used in the food preservation process. Bioaccessibility is the proportion of nutrients that the food matrix emits during the digestion process and can be absorbed, whereas bioavailability is the proportion of the former that enters the systemic circulation and is utilized by the body (162). This is a key difference because high bioaccessibility does not always imply high bioavailability because of physiological and metabolic limitations. The development of omics technologies has transformed the method of assessment, shifting away from conventional animal research to advanced *in vitro* and cellular models that can offer mechanistic details of the absorption mechanisms (163). *In vitro* model of digestion has been found essential in the assessment of nutrient bioaccessibility. The INFOGEST protocol is an internationally adopted protocol that can be used to assess the release of nutrients in complex matrices in an oral, gastric, and intestinal phase, allowing reproducible results. These models have been used to study the impact of non-thermal processing methods like high pressure processing, pulsed electric fields and ultrasound on the release of vitamins, minerals and bioactives in fortified foods (164). Omics based bioavailability analysis is a paradigm shift whereby metabolomics, proteomics and transcriptomics are integrated to effectively analyze the fate of nutrients since ingestion all the way to systemic use. Post-fortified food plasma metabolomics are direct evidence of absorbed nutrients and metabolites, bioavailability and efficacy biomarkers (139). Lipidomics has been especially useful in the determination of fat-soluble vitamins and bioactive lipids, and has shown how the composition of food matrices and processing conditions affects the efficiency of absorption (165). Additionally, gut microbiome analysis in combination with metabolomics has also further shown that intestinal bacteria is involved in biotransforming fortified nutrients into more bioavailable products, which has enhanced the importance of microbiome profiles in personalized nutrition (166). Together, these integrated omics systems offer critical knowledge in the development of fortified foods to deliver nutrients in the most effective way, make them effective, and use them as a means of precision nutrition.

### 5.5 Safety and toxicity evaluation

The safety and toxicity analysis of the fortified foods prepared using non-thermal methods demand in-depth approached strategies that go beyond conventional toxicological endpoints. The omics methods have molecular level insights about possible adverse effects and long-term safety implications. The toxicogenomics has become an influential paradigm, combining genomics, transcriptomics, proteomics, and metabolomics to discover molecular evidence of toxicity at doses and exposure durations that would not result in apparent clinical manifestations (167). Transcriptomic analysis of different tissues and cell lines treated with fortified food extracts allows the identification of disturbed pathways, stressors and adaptive responses that are predictive of toxicity. Submission to non-thermal processed foods demonstrates less toxicologically relevant molecular change over thermal techniques, which supports their safety profile and biomarkers of monitoring (168). The safety assessment of the product requires the use of metabolite profiling, which provides a detailed chemical characterization and identification of dangerous products formed during the processing or storage of the product in question. Untargeted metabolomics identifies unexpected changes, degradation products or neo-products that were generated in the process of interactions between fortificants and matrices. Non-thermal processing methods including HPP and PEF produces considerably lower levels of process contaminants compared to conventional thermal processes and the preservation of nutrient integrity (169). The prediction of allergenicity is gaining more relevance with new proteins and bioactives being added to the fortification. Proteomic studies define protein profiles, which allows the identification of allergens and prediction of cross-reactivity with the current food allergies (157). Safety studies demand monitoring biomarker panels and surveillance systems that have the ability to detect subtle adverse effects in populations during the long run. Longitudinal omics research indicate time related metabolic variations, cumulative effects and personal vulnerable factors that are not apparent in brief trials. Systems toxicology can be described as a merger between multi-omics datasets and physiological parameters and clinical outcomes to develop predictive models of long-term safety (170). The merging of omics technologies and conventional toxicology offers the most coherent platform to guarantee the safety of non-thermal fortified foods, as well as contribute to the emergence of customized nutrition tools.

## 6 Pathways to personalized nutrition

Personalized nutrition represents a paradigm shift of conventional one-size-fits-all dietary advice to individualized interventions that combine genetic, metabolic, microbiome, lifestyle, and health information and optimize their results and prevent disease (23). This precision strategy will be applied at both ends of the hierarchical spectrum, population-based interventions to subgroups that share common genetic variations or metabolic phenotype, and finally, to individualized dietary plans informed by multi-omics profiling. The combination of omics technologies and non-thermal food fortification allows the creation of tailor-made fortified foods, which retain bioactive compounds but fulfill particular nutritional needs (18). However, there are ethical and regulatory concerns in the application, including the fair access, the risk of genetic discrimination, and the rigidity of the evidence standards. Data privacy and security are of primary importance because personalized nutrition relies on sensitive health and genetic data, so effective measures are required to protect individual privacy and facilitate the responsible exchange of data in research and clinical translation (171).

### 6.1 Omics-based personalization

The integration of omics technologies has developed various ways through which molecular knowledge could be translated into dietary advice, essentially altering nutrition approach of a population-wide to an individualized approach. Omics-based personalization is a mixture of genomic, metabolomic, proteomic, and microbiome that concentrates on the multidimensional interplay of genetic predisposition, metabolic phenotype, and environmental conditions that influence individual responses to dietary constituents (85). This multi-omics implementation enables accuracy nutrition plans which considers inter-individual variations in nutrient metabolism, absorption and utilization and is no longer based on the one-size-fits-all nutritional guidance. The most comprehensive method is the consolidation of various omics methods with systems biology tools which integrates information of genomics, transcriptomics, proteomics, metabolomics, and metagenomics to understand the interactions of genes and their environment and metabolic networks (172). Digital health technologies, such as AI applications, CGM and wearable enable real-time physiological surveillance and dynamism in nutrition, converting multi-omics complexity into actionable and sustainable nutritional intervention (173). The combination of these holistic features, which is reinforced by the superior computational frameworks and digital provisions systems enables the development of non-thermal food fortification strategies and individualized nutrition solutions that are best suited to enhance health outcomes of various populations.

### 6.2 Digital health technologies

The combination of the digital health technologies and the personalized nutrition provided a paradigm shift in the principles of dietary intervention as they allow tracking in real time, data-driven perception, and an individual nutrition plan. The mobile apps and wearables are now the foundation of this ecosystem, which makes it possible to monitor diet intake, exercise routine, sleep pattern, and metabolism rates, which when combined, inform personal diets (174). Continuous glucose monitoring (CGM) systems are a type of radically new technology of personalized nutrition that provides individuals with an understanding of how individual responses to various foods and meal compositions affect glycemia at a granular level. CGM devices offer dynamic glucose profiles, which show postprandial excursions, nocturnal glucose changes, and the accumulated glycemic impact of dietary patterns during extended intervals, as compared to traditional point-in-time glucose measurements. CGM combined with machine learning algorithms has enabled the formation of predictive models to anticipate the glycemic response to novel food combinations and, therefore, to optimize the diet beforehand in case of a person with diabetes, prediabetes, or metabolic syndrome (175). Machine learning and artificial intelligence have emerged as powerful tools of promoting precision nutrition, and by analyzing extensive amounts of varied data like dietary history, omics profiles, CGM, and lifestyle data, actionable dietary guidance can be produced to address a specific phenotype and health objectives (176).

The AI-based applications make use of advanced algorithms like neural networks, random forests, gradient boosting machines, and so on to detect rich patterns and interactions among dietary factors, genetic variations, gut microbiome structure as well as metabolic outcomes that would otherwise be inaccessible using traditional statistical methods. Such systems can create personalized nutrition protocols that optimize nutrient intake and take into account personal preferences, cultural concerns, food allergies and budget constraints, which raise adherence and long-term success of dietary nutrition (174). Furthermore, the concept of digital biomarkers has gained prominence, to which new health indicators based on wearable sensors, smartphone applications, and connected devices can be included, which can continuously and objectively determine nutritional status, metabolic health, and dietary adherence. The smooth integration of these technologies is in the form of complete digital health bundles, which can bridge the gap between the knowledge of clinical nutrition and daily life with nutrition and democratize access to personalized nutrition services as well as generate rich datasets to further our understanding of diet-health relationships in diverse populations (177).

### 6.3 Personalized fortified foods

Integrated omics technologies are facilitating several convergent approaches to personalized nutrition that integrate non-thermal fortification technologies with personalized delivery systems, to provide viable ways from genomic and metabolomic data to customized nutrient interventions. On-demand fortification systems use modular dispensers and programmable cartridges to add micronutrients at the point of service or consumption depending on their genomic and metabolomic profiles, thereby preventing the exposure of heat-sensitive bioactives to thermal stress, and enabling real-time personalization as recommended by nutrigenomic models (178). Coaxial and gel-in-gel printing architectures can be used to preserve probiotic viability up to 90% during printing and 80% after simulated digestion, demonstrating the superiority of non-thermal processing. Smart packaging technologies integrate sensor-based reservoirs and moisture-stable compartments that deliver encapsulated nutrients based on time, temperature, or biochemical signals and is formulated and released profiles based on microbiome composition and metabolic risk measurements based on omics data (179). Among these pathways, micro-encapsulation of labile compounds, printable protein- and polysaccharide-based carrier matrices, coaxial architectures of compartmentalized delivery, sensor-integrated packaging of conditional release are all important technological mechanisms that have been shown to enhance bioavailability, dose accuracy, and targeted delivery in recent literature (180).

Combining nutrigenomic algorithms with these non-thermal fortification systems generates prescriptive actionable that can be implemented by cartridge dispersion, printed meals, or intelligent packages and simplifies the personalization of the system and introduces potential benefits including improved probiotic survival, accurate dosing of micronutrients and even changes the microbiota composition of an individual (181). Different pathways have different statuses of implementation, with 3D printing and on-demand systems moving to clinical and institutional demonstrations, subscription services going to pilot trials with consumer groups, and smart packaging being largely experimental, and all pathways are experiencing widespread challenges in scaling omics pipelines, standardizing non-thermal fortification procedures, creating regulatory frameworks around nutrient claims and release mechanisms, and producing solid evidence of safety, efficacy, and long-term health outcomes to move integrated omics and fortification technologies into widely accessible personalized nutrition solutions (182).

### 6.4 Clinical applications

Integrated omics strategies have led to a new era of precision in personalized nutrition intervention that is able to control disease prevention, therapeutic management, and nutritional optimization in relation to life stages with the highest level of accuracy. Precision nutrition systems are using multi-omics data to create specific dietary and nutritional plans to consider the genetic predisposition, metabolic phenotypes, and microbiome composition, and shift overall population-focused recommendations to individualized risk reduction regimens in the context of disease prevention (183). The combination of these omics-directed strategies with non-thermal food fortification technologies has a specific potential to maintain bioactive compounds mediating these protective effects, yet an implementation framework needs to weigh the precision strategies with population-based strategies to provide equal access and reach large populations with their health benefits (184).

Omics biomarker-guided therapeutic nutrition interventions have shown a real clinical outcome in randomized controlled trials, and nutrigenomics-based dietary counseling has been shown to substantially improve long-term adherence to intervention compared to conventional lifestyle intervention a key predictor of treatment success in the management of chronic diseases. The PREVENTOMICS trial is an example of such translational paradigm, which uses metabolomic and genetic clustering algorithm to create a customized meal plan to weight loss and reduce cardiometabolic risk and prove feasible clinical workflows which can be scaled using digital health platform and e-commerce delivery systems (185). The biomarker-guided interventions have resulted in the alteration of metabolic parameters glycemic control and lipid profiles as well as inflammatory markers, but the heterogeneity of the trial designs and outcomes measures highlights the necessity to conduct more conclusive studies with standardized endpoints to establish the clinical efficacy in diverse populations and disease conditions. Integrated omics applied to optimizing sports nutrition and maternal-fetal nutrition is conceptually interesting yet under-explored clinically in personalized nutrition. Nutrigenomic systems suggest athlete-specific proportions of macronutrients, antioxidant supplement, and recovery plans, which may lead to maximum performance and minimum injury by developing individualized dietary plans. Likewise, high-resolution nutrition in pregnancy and lactation can be beneficial in maximizing maternal health and fetal programming using customized micronutrient fortification and dietary counseling based on maternal omics phenotypes, and future applications of non-thermal fortification technologies to perinatal cohorts have a severe gap in the literature (183). The application of non-thermal food fortification in combination with these omics-based nutrition-related solutions is also associated with a chance to achieve nutritional integrity and enhance bioactive compounds delivery.

## 7 Technological challenges and solutions

There are several technological challenges to the implementation of non-thermal fortification using an integrated omics approach, which will have to be resolved to provide scalable products with high nutritional content and acceptable sensory characteristics. Large scale achievements with HPP, PEF and other non-thermal modalities show nutrient retention and microbial inactivation but display scale-up barriers in capital investment and process standardization which raise the costs of operations and create complexities in energy budgets required to adopt the technology in the industry (186). Therefore, empirical solutions focus on hybrid processing to focus efficacy and reduce energy requirements, modular equipment constructions to permit incremental scaling, and life-cycle evaluations to determine cost- and energy-effective paths as suggested to wider commercialization and technology maximization (187). Unlike thermal methods, non-thermal methods tend to reduce thermal-induced damage to vitamins, phytochemicals, and certain approaches such as ultrasound and cold plasma may cause oxidative reactions or enzyme changes that weaken shelf life and bioactivity (188). In order to counter such effects, targeted encapsulation, use of controlled atmospheres, antioxidant co-formulation and optimized exposure parameters, have been suggested as means of maintaining labile compounds and prolonging functional shelf-life (36). Sensory quality is another key issue since customers demand taste, color and texture matches, non-thermal treatment tends to maintain sensory qualities better, although it may cause changes in microstructure or surface characteristics, which influence mouthfeel and appearance, thus process optimization through sensory and consumer testing is the way to go before scale-up (186). Lastly, harmonization of regulatory acceptance and labeling is also not trivial and therefore variability in controlling validation and low harmonizing approval routes slows penetration; hence multidisciplinary interaction of researchers, regulators and industry to create standardized validation systems, safety dossier and unambiguous labeling guidelines is consequently proposed in present reviews. At the same time, specific cost–benefit, and the analysis of life-cycle can help to explain the trade-offs between the capital investment and long-term energy savings, and consumer education and program of the cycle repeat sensory evaluation can justify the acceptance in the market and reliability in the regulations (117). This kind of action will increase validated, omics-based fortified products up into regulated markets, governed and monitored by stakeholders.

## 8 Future prospectives

The combination of the next-generation omics technologies, artificial intelligence, and advanced-biotechnology-based platforms is expected to transform the approach of personalized nutrition and food fortification. Multi-omics and systems-level analytics are already becoming high-throughput in order to implement precision nutrition frameworks, which require the incorporation of big data and machine learning. The combination of nuclear magnetic resonance and other high-throughput physicochemical omics techniques with machine learning providing actionable food composition, biomarker of intake, and kinetic profile information can be utilized to inform non-thermal functionalization and quality control in fortified products. Precision fermentation, genome engineering and cultivated systems are examples of advanced biotechnology methods that can be used to supplement non-thermal objectives in the generation of concentrated micronutrient-rich products and scale-based production of novel proteins. Complex omics, microbiome and behavioral inputs will be operationalized by artificial intelligence and machine learning into individual recommendations and adaptive fortification plans. Precision medicine will interact with novel food sources and biotech platforms to form nutrigenomic and pharmaconutrition pathways that can be used in clinical care. Precision fermentation proteins, algae-derived nutrients, fermented functional foods, and cultured meat expand the array of bioavailable micronutrients and bioactives, which can be customized to an individual’s genetics and metabolism. Translational translational value Multi-omics and AI pipelines have already been shown to be useful in oncology nutrition and metabolic disease to support predictive algorithms and integrated workflows to the clinic in the future that may include fortified functional foods as an adjunct treatment. To achieve clinical adoption, research should focus on standardized assays, harmonized data models, and prospective clinical trials to show efficacy, safety as well as cost-effectiveness in different populations.

## 9 Conclusion

The convergence of integrated omics approaches with non-thermal food fortification technologies represents a transformative paradigm shift in nutrition science, offering unprecedented opportunities for personalized nutrition solutions that address individual metabolic phenotypes and health trajectories. Multi-omics platforms encompassing genomics, epigenomics, metabolomics, proteomics, and microbiomics have matured as the scientific backbone for precision nutrition, enabling deep phenotyping and tailored dietary interventions that surpass traditional one-size-fits-all recommendations. When coupled with non-thermal processing technologies that preserve bioactive compounds and enhance nutrient bioavailability, these advances may contribute to improved strategies for the design, delivery, and personalization of fortified foods, subject to further validation. However, realizing this vision requires addressing substantial implementation challenges, including methodological standardization across omics platforms, development of robust data integration infrastructure, reduction of costs for population-scale deployment, and establishment of clear regulatory frameworks for omics-driven nutritional claims. The path forward demands coordinated action from multiple stakeholders: the research community must prioritize biomarker validation through large-scale longitudinal studies and develop validated machine-learning algorithms; industry must invest in translational pipelines that convert omics insights into accessible fortified products and digital decision-support tools; policymakers must create governance structures that balance innovation with privacy protection and equitable access; and consumers require transparent education to build trust and appropriate expectations. The ultimate vision integrates personalized nutrition seamlessly within the broader health ecosystem, interoperable with electronic health records and preventive care systems, enabling both individual empowerment and population-level health improvement. This integration represents not merely technological advancement but a fundamental reimagining of nutrition’s role in precision medicine and public health, with profound implications for disease prevention, healthy aging, and societal well-being across diverse populations worldwide.
